# Submicromolar copper (II) ions stimulate transretinal signaling in the isolated retina from wild type but not from Ca_v_2.3-deficient mice

**DOI:** 10.1186/s12886-020-01451-8

**Published:** 2020-05-06

**Authors:** Jan Niklas Lüke, Felix Neumaier, Serdar Alpdogan, Jürgen Hescheler, Toni Schneider, Walid Albanna, Isha Akhtar-Schäfer

**Affiliations:** 1 Institute for Neurophysiology, University of Cologne, Robert-Koch Str. 39, D-50931 Cologne, Germany; 2 Department of Neurosurgery, University Hospital, RWTH Aachen, Aachen, Germany

**Keywords:** Murine ERG, Isolated vertebrate retina, B-wave, Reverse inhibition

## Abstract

**Background:**

So far, only indirect evidence exists for the pharmacoresistant R-type voltage-gated Ca^2+^ channel (VGCC) to be involved in transretinal signaling by triggering GABA-release onto ON-bipolar neurons. This release of inhibitory neurotransmitters was deduced from the sensitivity of the b-wave to stimulation by Ni^2+^, Zn^2+^ and Cu^2+^. To further confirm the interpretation of these findings, we compared the effects of Cu^2+^ application and chelation (using kainic acid, KA) on the neural retina from wildtype and Ca_v_2.3-deficient mice. Furthermore, the immediately effect of KA on the ERG b-wave modulation was assessed.

**Methods:**

Transretinal signaling was recorded as an ERG from the superfused murine retina isolated from wildtype and Ca_v_2.3-deficient mice.

**Results:**

In mice, the stimulating effect of 100 nM CuCl_2_ is absent in the retinae from Ca_v_2.3-deficient mice, but prominent in Ca_v_2.3-competent mice. Application of up to 3 mM tricine does not affect the murine b-wave in both genotypes, most likely because of chelating amino acids present in the murine nutrient solution. Application of 27 μM KA significantly increased the b-wave amplitude in wild type and Ca_v_2.3 (−|-) mice. This effect can most likely be explained by the stimulation of endogenous KA-receptors described in horizontal, OFF-bipolar, amacrine or ganglion cells, which could not be fully blocked in the present study.

**Conclusion:**

Cu^2+^-dependent modulation of transretinal signaling only occurs in the murine retina from Ca_v_2.3 competent mice, supporting the ideas derived from previous work in the bovine retina that R-type Ca^2+^ channels are involved in shaping transretinal responses during light perception.

## Background

Copper-, iron-, and zinc-ions are essential in human biochemical function. Their concentration in vivo is under tight control, and a dyshomeostasis may cause electrical imbalance and consecutively, region-selective neurodegeneration eventually facilitating cognitive deficits. In a systematic quantification of all three biometals in the human brain, age-associated changes in the elderly population were quantified in postmortem neocortical tissue. While Zn^2+^ was unaffected in any disease pathologies of the brain, both Cu^2+^ and Fe^3+^ showed a gradual age-associated decline in healthy non-cognitively impaired individuals. Further, Cu^2+^ was significantly reduced by 20%, and Fe^2+^ significantly increased by 10–16% in severe Alzheimer disease (AD) compared with age-matched controls [1].

Various ion channels were reported to be highly sensitive towards Zn^2+^ and Cu^2+^ at concentrations below 10 μM (summarized in [2, 3]). Among them are glutamate-, glycine-, GABA-A-, acetylcholine- and P2X- receptors as well as voltage-gated Na^+^ (Na_v_1.5), K^+^ (K2P, Kv1.3, mSlo1), and Ca^2+^ (Ca_v_3.2) channels [4–6].

Polyvalent inorganic cations can interfere with the function of VGCCs through various mechanisms, which include electrostatic effects, pore block, and in some cases allosteric effects [3]. Besides, endogenous Zn^2+^ and Cu^2+^ are increasingly recognized to be involved in central neurotransmission, although their exact role remains ambiguous. Both can be released spontaneously or in an activation-dependent manner from defined populations of neurons located primarily in limbic regions (hippocampus, amygdala) in the cerebral cortex and in the retina [7] (for a summary see [8]).

The isolated and superfused vertebrate retina represents an important model of a neuronal network for the functional investigation of postsynaptic excitation conduction [9–11]. Using the bovine retina, the process of reciprocal inhibition during transretinal signaling was investigated in detail [8, 12, 13]. Cu^2+^ occupy an allosteric binding site on the domain I gating module of Ca_v_2.3 channels and may interfere with voltage-dependent-gating. Several studies have shown that even very nominal Cu^2+^ concentrations are sufficient for significant suppression of these channels, as electroretinographically demonstrated on the ex-vivo bovine retina [14]. It was found that L-Glutamate, as excitatory amino acids, can stimulate Ca_v_2.3-channels by being as trace metal chelators and inhibiting the suppressing effects of metal ions such as Zn^2+^ and Cu^2+^ [2]. The naturally occurring excitatory amino acid Kainic acid has an embedded L-glutamic acid unit, and thus, a potent agonist at (non-NMDA) glutamate receptors. The systemic application produces epilepsy in rodent experiments [15] and in this context, Ca_v_2.3 channels can act to the pathogenesis of KA-induced seizures [16, 17]. However, the structural similarity of Kainat to L-Glutamate led us to investigate the chelating effect in the presence of physiologically concentrations of Cu^2+^.

To understand molecular mechanisms of the biometals, this technique of the isolated and superfused retina was successfully transferred to the isolated mouse retina from control and Ca_v_2.3-deficient mice [18, 19]. Those recordings of a full electroretinogram (ERG) from the isolated and superfused murine retina were optimized only recently [11] by modifying the composition of the standard Ames solution [20]. After gentle isolation of the retina, the development of the b-wave, indicative for transretinal signaling, was stabilized by adding barium chloride (0.1 mM) to the final perfusion solution, similar as it was reported originally for eyecup preparations of the tiger salamander [21].

In the present report, recordings of the full ERG were possible and were compared between the Ca_v_2.3-competent and the –deficient mouse retina to understand if Ca_v_2.3 is involved in transretinal signaling as supposed from recordings in the bovine retina.

## Methods

### Materials

Glucose and the constituents of the nutrient solution used for retinal superfusion were purchased from Merck (p.a. grade). For the preparation of the murine retina [11] a superfine scissor (WPI, Nr. 501,839), and ultrafine suturing forceps (WPI, Nr. 555063FT) were used. Further, a 27-gauge needle (Sterican, size 20: 0.4 mm × 20 mm Bl/LB) was used to punch a hole into the cornea of the extirpated eye bulb. The receptor antagonists UBP 301 and CNQX were purchased from Sigma Aldrich (Munic, Germany).

### Animals

In order to compare ERG responses from mice deficient of the voltage-gated Ca^2+^-channel Ca_v_2.3 (R-type), we used control mice with an identical genetic background. Both mouse lines were generated and bred in our animal facility. Meanwhile, they can also be ordered from MMRRC (ID 50523).

Ca_v_2.3-deficient animals and control mice were kept as separate mouse lines derived from heterozygous parents (fourth backcrossing into C57Bl/6). Homozygous littermates were regularly interbred with each other and back-bred into C57Bl/6 (for further information on Ca_v_2.3-deficient generation see [22, 23]). In short, the cacna1e gene encoding Ca_v_2.3 was disrupted in vivo by agouti-colored Ca_v_2.3(fl|+) and deleter mice expressing Cre-recombinase constitutively [24]. Thus, exon 2 was ablated by Cre-mediated recombination. Ca_v_2.3-deficient mice were fertile, exhibited no obvious behavioral abnormalities and had the same lifespan as control mice. The Cav2.3-deficient mouse line, which was generated in the Cologne lab was transferred to the Mutant Mouse Resource & Research Centers (MMRRC) with the strain name B6J.129P2(Cg)-Cacna1e^tm1.1Tsch^/Mmjax.

Adult male mice were used at the age of 12 to 18 month and kept at 20 to 22 °C in makrolon type II cages under a 12 h light-dark cycle (7:00 a.m./p.m.) with food and water provided ad libitum*.* All animal experiments were in line with the European Communities Council Directive 2010/63/EU for the care and use of laboratory animals as described in the UFAW handbook on the care and management of laboratory animals. All experiments were approved by the local institutional committee on animal care (UniKöln_Anzeige§4.17.007).

### Methods

In order to reach maximum transretinal signaling realized by a full ERG, the bovine [10] and the murine retina [11] had to be superfused under different conditions (Table 1).

**Table 1:** Comparison of nutrient solutions used for ERG recordings from bovine (Sickel-medium) or murine isolated retina (modified AMES-medium). AMES-medium was modified by increasing the pH slightly to alkaline (pH 7.7) and by adding 0.1 mM BaCl_2_

Nutrient	Sickel-medium	modified AMES-medium
NaCl	120 mM	120 mM
KCl	2 mM	3.1 mM
CaCl_2_	0.15 mM	1.15 mM
MgCl_2_/MgSO_4_ * 7 H_2_O	0.1 mM	–
D-glucose	5 mM	6 mM
MgSO_4_	–	1.24 mM
KH_2_PO_4_	–	0.5 mM
NaH_2_PO_4_	1.5 mM	–
Na_2_HPO_4_	13.5 mM	–
NaHCO_3_	–	22.6 mM (pH 7.4)
		45.3 mM (pH 7.7)
L-glutamine, amino acids, vitamins and other components	–	Sigma-Aldrich Nr. A1420
Equilibration	Pure oxygen	Carbogen gasing containing 5% (v/v) of CO_2_ in O_2_
pH	7.8	7.4–7.7
BaCl_2_	–	0.1 mM

Bovine eyes were received from a local slaughterhouse and immediately stored in a nutrient solution (Sickel medium), which was aerated with pure oxygen, consisting of the following (in mM): NaCl (120), KCl (2.0), CaCl_2_ (0.15), MgCl_2_ (0.1), NaH_2_PO_4_ (1.5), Na_2_HPO_4_ (13.5) and glucose (5) with a final pH of 7.8. The bovine retina was isolated as described in detail by Luke et al. 2005 [10]. After isolation, a plain retina segment was transferred into the recording chamber described below, which is placed in an electrically and optically isolated air thermostat. From the dark-adapted retina and in response to a single white flash, electroretinograms were recorded at intervals of 5 min at 30 °C, with a constant superfusion at 1 ml/min controlled by a roll pump. The flash intensity was set to 6.3 mlux, with the duration of light stimulation at 500 ms [10].

After reaching a stable equilibrium of the light-evoked ERG responses, 5 mM tricine (tricine, Sigma # RES3077T-A701X) was added to the nutrient solution with 10 mM (HEPES) (Carl Roth, p.A., # H3375) and superfused for 30 min. Washout was started thereafter with Sickel medium.

Murine retinas were isolated from mice of our animal facility department, in which the light–dark regime was 12:12 h, and the light intensity between 5 and 10 lx at the surface of the animal cages.

DNA-containing tissue samples were collected from tail biopsies. DNA was extracted and used as template for genotyping. Transcripts of Ca_v_2.3 were detected by RT-PCR (RT, reverse transcriptase) using primers, which flanked the deleted exon 2 and exon 3 region [22]. In short, contaminating protein and RNA were enzymatically digested by protease and RNAse, respectively. For the PCR amplification of indicative Ca_v_2.3 DNA-fragments, about 1 μg DNA was introduced and amplified with the forward primer (B45Hilx1) 5′- AAA AAC AGC CGG GGA AAG CTT AT-3′ and the reverse primer (a1eb45r) 5′-CTG CCC TTT CTT CTT GCC TGA C-3′. The sizes of DNA fragments expected are 1056 bp for the WT and 86 bp for the Ca_v_2.3-KOs. PCRs for the genotyping were performed using a DNAEngine Peltier thermal cycler (BioRad, Germany) or a PTC-200 Peltier thermal cycler (MJ Research, Biozym Diagnostik, Germany) with the initial denaturation (94 °C for 10 min) followed by 34 cycles (denaturation at 94 °C for 60 s, annealing at 60 °C for 90 s, extension at 72 °C 4 min) and final extension at 72 °C for 10 min. The PCR products were separated by agarose gel electrophoresis and fluorescent bands were detected on a Herolab UVT-28 M transilluminator by UV irradiation (312 nm excitation wave length).

For Ca_v_2.3, mouse lines were used as separate inbred strains for Ca_v_2.3(+|+) and Ca_v_2.3(−|-), each after the fourth backcrossing in C57Bl / 6 mice.

The mice used for the retina isolation were dark adapted overnight, sacrificed by cervical dislocation under dim red light, without the need for an anesthetic, and the eyes were extirpated immediately. Enucleated eyes were protected from light and transferred into carbogen (95% O_2_ / 5% CO_2_)-saturated modified Ames medium respectively [11]. The isolation of the murine retina was started immediately post mortem and carried out under dim red light. The complete retina was transferred to the recording chamber [18] and the electroretinogram was recorded via two silver/silver-chloride electrodes on either side of the isolated retina. The recording chamber containing the retina was placed in an electrically and optically isolated air thermostat. From the dark-adapted retina responses to a single white flash were recorded at intervals of 3 min at 27.5 °C and with a constant superfusion at 2 ml/min controlled by a roller pump. The duration of light stimulation was 500 ms, controlled by a timer operating a mechanical shutter system. The pre-stimulus delay was 380 ms. Unless noted otherwise, the flash intensity was set to 63 mlux at the retinal surface using calibrated neutral density filters.

As soon as the isolated retina was placed into the recording chamber, it was equilibrated for about 60 min in modified Ames-solution (*n* = 20 ERGs) (Table 1). After reaching a stable equilibrium of the light-evoked ERG responses, 100 nM CuCl_2_ was added to the modified Ames-medium and superfused for 30 min (*n* = 10 ERGs). Washout was started thereafter with Cu^2+^-free modified Ames-medium. Each ERG response contains 239 data points.

The ERG was amplified and bandpass-limited between 0.3 and 300 Hz (PowerLab 8/35; Animal Bio Amp FE136, ADIntruments, Oxford, UK). Light flash, heating unit, fan and roller pump were automatically controlled by National-Instruments (BNC-2120; DASY-Lab V8.0). For each experiment, a new retina was transferred to the recording chamber. The retina was superfused with nutrient solution and stimulated repetitively until the responses had reached a stable level (usually after 60 min of perfusion). Switching from one solution to another was performed with a three-way valve to prevent disturbance of the experimental conditions. Experimental protocols for the isolation, storage and incubation of the vertebrate retina can markedly alter phototransduction and transretinal signaling [25]. To quantify such changes, we evaluated the a- and b-wave amplitudes and their implicit times.

### Data analysis

The b-wave amplitude was measured from the trough of the a-wave to the peak of the b-wave. After reaching the equilibrium, the initial b-wave amplitude was set to 100% (= Pre) resulting preferably illustration of ERGs after treatment with drugs as well as after washout. Quantitative normally distributed data are presented as mean ± standard error of mean (SEM) and as percentage. Nonparametric tests are demonstrated as median [1. quartile – 3. quartile]. Two-sided, paired Student t-test was used for comparison of quantitative parameters in case of normal distribution. If not applicable, Wilcoxon test was used instead.

All analyses were performed with IBM® SPSS® Statistics V22.0 (IBM, Chicago, Illinois, USA).

## Results

### Trace metal chelation by tricine in the bovine and the murine retina

The bovine retina was adapted to a phosphate buffered nutrient solution (“Sickel-medium”), which was aerated with pure oxygen and contained a reduced Ca^2+^ concentration to prevent precipitation of calciumphosphate (apatit).

As described in a previous publication, stable ERGs from the murine retina can be recorded in a carbonate buffered nutrient solution (AMES-medium), comprising additionally several amino acids. Further, the medium was slightly modified by adding 0.1 mM BaCl_2_ to avoid the M-wave causing a reduction of the apparent b-wave amplitude [21] and by shifting the pH to a more alkaline value (Table 1).

**Fig. 1:**
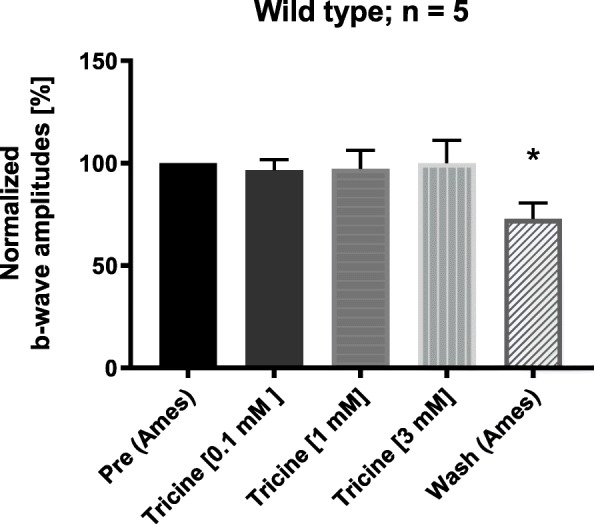
Comparison of the normalized b-wave amplitudes from five isolated retinas (*n* = 5 mice) after successive application of increasing concentrations of tricine and its washout. The initial b-wave amplitude, after reaching the equilibrium in Ames-medium, was set to 100% (= Pre). Tricine did not affect the ERG b-wave amplitude compared to the initial amplitude in equilibrium (0.1 mM tricine: 96.5 ± 5,2%; 1 mM: 97.3 ± 9.0%; 3 mM: 100.1 ± 11.1%). After washout (= Post) the amplitude was significantly reduced to 72.8 ± 7.7%. *p < 0.05

The ERG b-wave amplitude of the bovine retina is known to be reversibly reduced in the presence of 5 mM tricine [14], which was shown to be caused by the chelation of trace metal cations as impurities in HEPES buffer or by chelation of 100 nM added CuCl_2_. In the bovine retina, submicromolar Cu^2+^ [14] blocks the Ca_v_2.3 R-type Ca^2+^ channel even more effectively than comparable NiCl_2_ [12] or ZnCl_2_ concentrations [8]. However, tricine did not change significantly the b-wave amplitude of the murine retina during successive application of 0.1, 1 and 3 mM tricine in the superfusing solution (Fig. 1, *n* = 5). During washout of the highest concentrations, a significant reduction of the b-wave amplitude to 72.8 ± 7.7% was observed, which may be the result of the longer lasting procedure of successive application of increasing tricine concentrations. The lack of b-wave reduction following the application of tricine in the murine retina could be explained by the presence of L-glutamate (L-Glu) in the Ames solution, because L-Glu and other amino acids are known to chelate divalent cations similar to tricine [2].

### Stimulation of transretinal signaling by copper (II) ions in Ca_v_2.3 competent mice

**Fig. 2:**
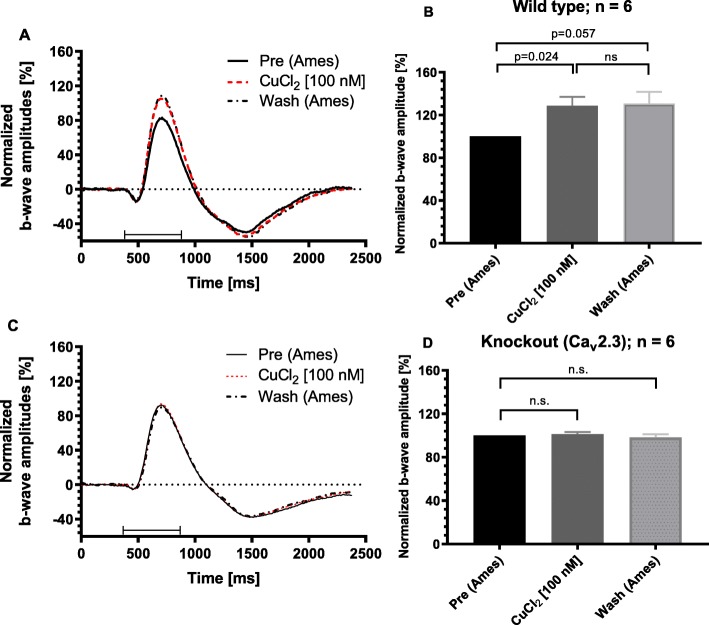
Changes of the b-wave amplitude after application of nominally 100 nM CuCl_2_ for control and Ca_v_2.3-deficient mice. **a** Mean representative ERG traces (*n* = 3 consecutive sweeps) for a single retina from Ca_v_2.3-competent mice before, after 30 min of superfusion with 100 nM CuCl_2_ in modified Ames-medium as well as after 30 min of washout. **b** Normalized mean values for retinas (*n* = 6 separate retinas) from Ca_v_2.3-competent mice before and after the superfusion with 100 nM CuCl_2_ as well as after 30 min of washout**.** The b-wave amplitude was significantly increased by 28.8 ± 8.2% (*p* = 0.024) in Ca_v_2.3-competent mouse retinas. **c** Mean representative ERG traces (*n* = 3 consecutive sweeps) for a single Ca_v_2.3-deficient retina before and after 30 min of superfusion with 100 nM CuCl_2_ in modified Ames-medium as well as after 30 min of washout. Note, that in comparison to the competent mice no change of the b-wave amplitude was observed (all three traces line up together). **d** Normalized mean values for retinas (*n* = 6 separate retinas) from Ca_v_2.3-deficient mice (Knockout) before and after the superfusion with 100 nM CuCl_2_ as well as after 30 min of washout. Note, that in comparison to the Ca_v_2.3(+|+) mice no significant change of the b-wave amplitude was observed. The b-wave amplitude following stimulation with 100 nM CuCl_2_ was 101.3 ± 1.9% (*n* = 6)

**Fig. 3:**
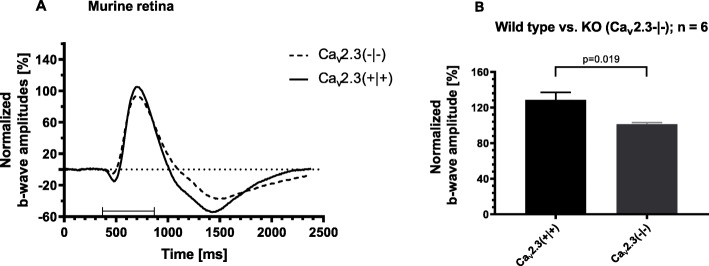
Comparison of the ERG b-wave amplitudes and shape of the ERG traces from both murine genotypes. **a** Representative ERG tracers from wild type and Ca_v_2.3-deficient mice, 30 min after superfusion with 100 nM CuCl_2_. **b** After 30 min of superfusion with 100 nM CuCl_2_ the b-wave amplitude is significantly increased to 129% ± 8% (*n* = 6) for the retina from wild type but not from Ca_v_2.3-deficient mice (KO, Knockout) (101% ± 2%, *n* = 6). Note, the shape of the ERG traces from both genotypes differ substantially shortly after the end of the triggering light flash

Since adding tricine to the perfusion solution had no effect on the ERG b-wave, we concluded that L-Glu from the Ames solution chelated any impurities from the nutrient solution additives, resulting in different signals when compared to recording of the bovine retina [14]. Next, the scotopic full ERG was recorded from six independent freshly isolated retinae either from control or from Ca_v_2.3-deficient mice (first eye only). After 30 min of superfusion with nominally 100 nM CuCl_2_ the b-wave amplitude was significantly increased by 28.8 ± 8.2% (*p* = 0.024, *n* = 6) in Ca_v_2.3-competent mouse retinas. This increase in b-wave amplitude remained irreversible during the subsequent washout (Fig. 2a and b). No change of the b-wave amplitude was observed in the ERG b-wave responses from Ca_v_2.3-deficient mouse retinae (Fig. 2c and d). The b-wave amplitude following stimulation with 100 nM CuCl_2_ was 101.3 ± 1.9% (*n* = 6) and was significantly lower (*p* = 0.019, *n* = 6) than in the Ca_v_2.3-competent controls (Fig. 3).

In a previous study, the isolated bovine retina exhibited a similar sensitivity towards 100 nM CuCl_2_ as the retina from wild type mice (for details [14]).

Taken together, these results suggest that submicromolar CuCl_2_ affects transretinal signaling via the Ca_v_2.3 VGCC in the wild type murine and probably also in the bovine retina.

### Multiple effects of kainic acid (KA) in the murine retina

**Fig. 4:**
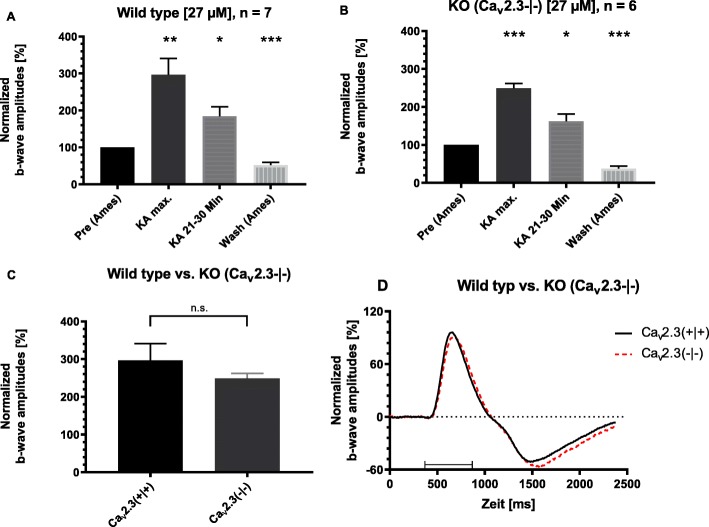
Effect of kainate superfusion on the ERG b-wave amplitudes from both genotypes. After reaching the equilibrium of a maximum b-wave amplitude without kainate (KA), the nutrient solution was completed with 27 μM KA causing a transient increase of the b-wave amplitude. For each genotype, the initial amplitude (“Ames”) was plotted as 100%. The subsequent maximum amplitude (“KA max.”) and the amplitude at the end of the 30 min superfusion period (“KA 21/30 Min.”) was plotted as relative increase, respectively. During washout, the amplitude decreased beyond the initial equilibrium value. *p < 0.05, **p < 0.01, ***p < 0.001. **a** Normalized amplitudes of the retinas from Ca_v_2.3/competent mice (*n* = 7). During the 30 min superfusion period, an increase of the b-wave occurred in Ca_v_2.3-competent mice to 297 ± 44% of the initial amplitude in equilibrium (p < 0.01). The b-wave amplitude was still significantly (*p* < 0.05) increased at the end of the KA washing in period (184 ± 5%). After the washout, the b-wave amplitude was significantly reduced (*p* < 0.001). **b** Normalized amplitudes of the retinas from Ca_v_2.3-deficient mice (KO, Knockout) (*n* = 6). In Ca_v_2.3-deficient mice, the b-wave increased to 249 ± 13% (*p* < 0.01). The b-wave amplitude was still significantly (*p* < 0.05) increased at the end of the KA washing in period (162 ± 19%). After the washout, the b-wave amplitude was significantly reduced (*p* < 0.001). **c** Comparison of the maximum values from panel A and B for both genotypes. The maximum increase was not significantly different between both genotypes (see **b**). **d** Overlay of the mean traces for wild type and Ca_v_2.3-deficient mice

For the human Ca_v_2.3 R-type Ca^2+^ channel, a reciprocal modulation by Cu^2+^ and kainic acid was found when stably co-expressed with human β_3_-subunit in HEK-293 cells. Moreover, when tricine was used as a surrogate of kainic acid, it produced effects consistent with Ca_v_2.3 channel modulation both in transfected HEK cells and in the isolated bovine retina [14]. In the murine retina, tricine was ineffective in changing transretinal signaling, likely due to the trace metal chelation by glutamate which was present in the Ames medium. We therefore analyzed the effects of direct administration of kainic acid at the same maximal concentration that was used in the heterologous system [14] by adding 27 μM kainic acid to the Ames perfusion solution (Fig. 4). It caused a fast and highly significant increase of the ERG b-wave amplitude in retinas from both genotypes (Fig. 4a and b). During the 30 min superfusion period, a fast increase occurred in Ca_v_2.3-competent mice to 297 ± 44% of the initial amplitude in equilibrium and in Ca_v_2.3-deficient mice to 249 ± 13% within the first 12 min (both increases were highly significant, *p* < 0.01, *n* = 7 (Ca_v_2.3-competent mice) and *n* = 6 (Ca_v_2.3-deficient mice)). The b-wave amplitude was still significantly (*p* < 0.05) increased at the end of the KA washing in period in Ca_v_2.3-competent mice (184 ± 5%) and Ca_v_2.3-deficient mice (162 ± 19%). In both genotypes, the b-wave amplitude was significantly reduced (*p* < 0.001) after washout of KA (Fig. 4a and b) compared to the initial b-wave amplitude. The maximally observed increases during the beginning of KA superfusion did not reach the level of significance, when comparing both genotypes (Fig. 4c). KA does not have a prominent and visible effect as a chelating agent under the conditions for recording murine ERGs, since no reduction of the ERG b-wave amplitude was observed.

**Fig. 5:**
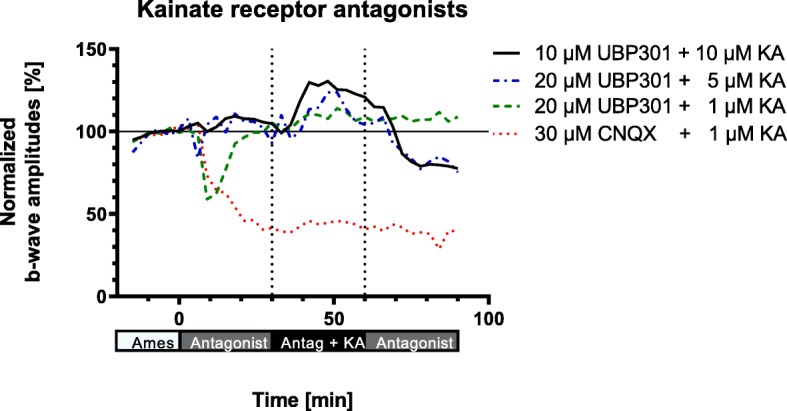
Effect of KA-receptor antagonists on the KA-mediated increase of the ERG b-wave amplitudes from Cav2.3-competent mice. Normalized amplitudes were plotted under the conditions as indicated. KA was added in the presence of the KA-receptor antagonists. Note, only under 30 μM CNQX the amplitude increase was completely prevented

The next series of experiments were performed to block the native ionotropic kainate receptors in the murine retina, in order to unmask a potential chelating effect of KA during transretinal signaling. Two antagonists were used to inhibit the stimulatory effect of KA on its receptors (Fig. 5). The high affinity broad spectrum kainate receptor antagonist UBP 301 [26] was used at 10 and 20 μM concentration with either 10, 5 or only 1 μM KA. However, all three concentrations did not prevent the KA-induced increase of the b-wave amplitude (Fig. 5). Using the competitive AMPA / kainate receptor antagonist CNQX (30 μM), which also antagonizes NMDA receptors at glycine sites, a reduction to less than 50% of the initial amplitude was observed. This effect remained unchanged even in the presence of KA (1 μM) leading to the conclusion that KA prominently activates ionotropic glutamate receptors in the murine retina. Thus, the chelating effect of KA cannot be visualized in the organotypic preparation of the isolated murine retina.

## Discussion

During transretinal signaling in the vertebrate retina, VGCCs are involved in basal synaptic signal transduction by triggering Ca^2+^-mediated glutamate release. In the dark, the excitatory neurotransmitter glutamate is tonically released from ribbon synapses and the rate of release is modulated in response to graded changes in the membrane potential contrasting with action potential–driven bursts of release at conventional synapses [27]. Mainly the two L-type VGCC containing the ion conducting subunits Ca_v_1.3 (α1D) and Ca_v_1.4 (α1F) appear to form the principle voltage-gated Ca^2+^ influx pathways in rods and cones [28], but also in the cochlear inner hair cells [29]. The importance of both Ca_v_1 L-type Ca^2+^ channels during sensory signaling gets obvious after its ablation, either by recombinant technologies for Ca_v_1.3, leading to deafness [30] or after native gene loss for Ca_v_1.4 leading to night blindness [31].

Transsynaptic signaling is modulated by GABA and glycine, and by additional neurotransmitters and other signaling molecules like nitric oxide (NO), acetylcholine and dopamine. So far, these synaptic modulation and integration has not yet been fully understood. For the bovine retina, it was postulated that the R-type Ca^2+^ channel may be involved in this reciprocal inhibition by triggering the release of inhibitory neurotransmitters [13]. As previously demonstrated in our experiments on the murine retina, such an involvement was deduced by recording and calculating the amplitude changes caused by Ni^2+^ application, a rather complicated indirect determination [19]. The inhibitory reciprocal modulation by GABA was related to the expression of both, Ca_v_2.3 / pharmacoresistant R-type and Ca_v_3.2 / T-type voltage-gated Ca^2+^ channels [19], which are both highly sensitive towards toxic Ni^2+^.

The ten different genes encoding the ion conducting subunits show different sensitivities towards bioavailable trace metal cations Zn^2+^ and Cu^2+^ [3]. Both, Ca_v_2.3- and Ca_v_3.2-channels are most sensitive towards Zn^2+^ and Cu^2+^ [2, 32]. Therefore, the sensitivity towards Cu^2+^, the most effective divalent metal cation inhibitor, was investigated first in the isolated and superfused bovine retina [14] and now in the retina of wild type and genetically modified mice lacking the expression of Ca_v_2.3. The present report provides further support to the idea that the assumed R-type Ca^2+^ channel containing Ca_v_2.3 as ion conducting pore is involved in transretinal signaling and modulated by submicromolar Cu^2+^ concentrations. This concentration mimics the in vivo situation and indicates that Cu^2+^ and probably also other bioavailable trace metal cations could help to modulate the strength of signal propagated through the retinal network in vivo.

Major differences between the Cu^2+^ effects on transretinal signaling, which will be discussed, are related to a genotype-related difference. The genotype-dependent stimulation in Ca_v_2.3-competent mice shows that submicromolar Cu^2+^ mediates its effect clearly via Ca_v_2.3, the pharmacoresistant Ca^2+^ channel, which, in the bovine retina, was thought to trigger GABA-release from amacrine cells onto ON-bipolar neurons. This release of inhibitory neurotransmitters was deduced from the sensitivity of the b-wave to stimulation by Ni^2+^ [12, 13], Zn^2+^ [8] and Cu^2+^ [14]. For the isolated murine retina expressing Ca_v_2.3, the same concentration of CuCl_2_ (100 nM) caused a similar effect, namely a significant 30% increase of the ERG b-wave amplitude, which was not observed in the retinae from Ca_v_2.3-deficient mice.

It should be noted that the bovine retina [10] was isolated and recorded in a different medium than the murine retina [11], although in both cases, the solutions were optimized to achieve a maximal b-wave response, indicative of a “healthy” retina. The major difference between both nutrient solutions is related to the absence (bovine) or presence (murine) of an amino acid cocktail including glutamate, which can chelate submicromolar concentrations of divalent trace metal cations. Therefore, control recordings by chelating Cu^2+^ after application of tricine failed for the murine retina (present report, Fig. 1) but not for the bovine retina [14]. Attempts to record from the murine retina under conditions previously used for the bovine retina (i.e. with Sickel instead of Ames medium) failed, possibly reflecting the higher metabolic needs of the murine retina.

Another set of experiments was related to the effects of kainate, which is routinely used to induce experimental seizures in mice. As this drug is also able to chelate trace metal cations, we tested its effects on transretinal signal transduction in the isolated retina. Kainic acid (KA) probably affects more efficiently the high-affinity binding sites of the classical non-N-methyl-D-aspartate (non-NMDA) ionotropic glutamate receptors, which are expressed in the murine retina [33, 34]. Under the conditions for recording murine ERGs in our setup, KA did not have a prominent and visible effect as a chelating agent, since no reduction of the ERG b-wave amplitude was observed. However, we failed to find any effect on the Cu^2+^ mediated stimulation, because endogenous kainate receptors could not be blocked completely by KA-receptor antagonists or the antagonists themselves showed pronounced effects on transretinal signal transduction (Fig. 5).

In summary, regardless of the exact mechanisms of Cu^2+^ effects, our findings show that genetic inactivation of Ca_v_2.3 channels is sufficient to prevent Cu^2+^-mediated stimulation on the b-wave amplitude. The results illustrate that Cu^2+^-induced responses in Ca_v_2.3 channel function can lead to changes in the normal neurotransmission. Based on our results using the isolated retina model and previous works [35, 36], an integrative model of Cu^2+^-depended cellular responses is presented. Therefore, the involvement of VGCCs could reflect a potential target for interventions of diseases as reported by several researchers [4, 37, 38]. Further investigation will be necessary to establish the relevance of trace metal ions on VGCCs.

## Conclusion

The present report demonstrates that submicromolar concentrations of the physiologically relevant trace metal ion Cu^2+^ cause an increase of the b-wave amplitude in retinae from wild type but not Ca_v_2.3-deficient mice, suggesting that the effect is mediated by suppression of Ca_v_2.3 channels. Based on previous studies in the bovine retina, Ca_v_2.3-triggered GABA release could activate chloride influx into ON-bipolar cells and thereby reduce the b-wave amplitude [13]. Thus, inhibition of Ca_v_2.3 by CuCl_2_ may reduce reciprocal inhibition via GABA receptors and would increase the b-wave amplitude, as shown here for the murine retina. Kainate did not show any visible effects as a chelator for ions on murine ERG, demanding advanced experimental studies to understand the mechanism more precisely. The described model may facilitate further investigation of trace metal ions on calcium channels to determine their exact role in several diseases.

## Data Availability

All data are available on the local backup computer and will be sent upon request.

## Abbreviations

Ca^2+^: Calcium ions
Ca_v_2.3: The ion conducting subunit of the pharmacoresistant voltage-gated calcium channel
CNQX: 7-nitro-2,3-dioxo-1,4-dihydroquinoxaline-6-carbonitrile, a competitive AMPA / kainate receptor antagonist
ERG: Electroretinogram
GABA: Gamma amino butyric acid
KA: Kainic acid
L-type: Subfamily of high-voltage gated calcium channels with four members (Ca_v_1.1 to Ca_v_1.4)
NMDA: N-methyl-D-aspartate
R-type: Member of the subfamily Ca_v_2 of the high-voltage gated calcium (Ca_v_2.3)
VGCC: Voltage-gated calcium channel
